# *Real‐World*‐Daten zu Tirbanibulin bei aktinischer Keratose in Deutschland ‐ Prospektive multizentrische nichtinterventionelle KLIR‐Studie

**DOI:** 10.1111/ddg.15876_g

**Published:** 2026-02-05

**Authors:** Markus V. Heppt, Ina Hadshiew, Afra Kempf, Antje Melzer, Carola Berking

**Affiliations:** ^1^ Hautklinik Uniklinikum Erlangen Friedrich‐Alexander‐Universität Erlangen‐Nürnberg, Erlangen; ^2^ Comprehensive Cancer Center Erlangen‐EMN, Erlangen; ^3^ Bayerisches Zentrum für Krebsforschung (BZKF), Erlangen; ^4^ MVZ Derma Köln, Köln; ^5^ Almirall Hermal Reinbek Deutschland

**Keywords:** Aktinische Keratose, nichtinterventionelle Studie, patientenberichtete Ergebnisse, *Real‐World*‐Evidence, Tirbanibulin, Actinic keratosis, non‐interventional study, patient‐reported outcomes, real‐world evidence, tirbanibulin

## Abstract

**Hintergrund:**

In dieser nichtinterventionellen Studie wurde die Wirksamkeit und Sicherheit von Tirbanibulin, einem zugelassenen topischen Medikament zur Behandlung aktinischer Keratosen (AK), untersucht.

**Patienten und Methodik:**

Die prospektive nichtinterventionelle Studie wurde an 58 dermatologischen Kliniken und Praxen in Deutschland durchgeführt (02/2022–09/2023). Die Teilnehmenden trugen Tirbanibulin einmal täglich an 5 aufeinanderfolgenden Tagen auf das von AK betroffene Hautareal auf. Nach 57 Tagen (Visite 3) wurden die Reduktion der AK‐Anzahl, die Abheilungsrate, die Verträglichkeit sowie die Behandlungszufriedenheit bewertet. Bei den weiteren Visiten wurden die Basisdaten (Tag 0, Visite 0), ein mögliches Maximum lokaler Hautreaktionen (LSR) (Tage 8–29, Visite 2) sowie die Rezidivrate (Tag 240, Visite 4) untersucht.

**Ergebnisse:**

Insgesamt nahmen 545 Patienten (68% männlich) mit AK an der Studie teil. Die durchschnittliche AK‐Anzahl sank um 70%, von 5,9 (Ausgangswert) auf 1,9 bei Visite 3 (*p* < 0,0001). Bei 37,4% der Teilnehmenden war bei Visite 3 eine vollständige Abheilung zu beobachten, bei 55,0% eine partielle Abheilung (≥ 75% Rückgang). Von den Patienten mit vollständiger Abheilung zeigten 21,9% bei Visite 4 erneut AK‐Läsionen. Die meisten LSR waren leicht bis moderat, wobei am häufigsten Erytheme (97,6%) auftraten. Insgesamt berichteten 91,5%, dass sich ihre AK‐Läsionen verbessert hätten, und 99% würden eine Behandlung mit Tirbanibulin in Zukunft wieder in Betracht ziehen („auf jeden Fall“ [45%], „sicher“ [28%], „wahrscheinlich“ [15 %], „vielleicht“ [11 %]).

**Schlussfolgerungen:**

Tirbanibulin zeigte eine hohe Abheilungsrate und Akzeptanz im medizinischen Versorgungsalltag.

## EINLEITUNG

Die aktinische Keratose (AK) ist eine häufige Hauterkrankung und kann in ein kutanes Plattenepithelkarzinom übergehen; sie entsteht überwiegend auf chronisch UV‐exponierter Haut.^1^, ^2^ In Europa liegt ihre Prävalenz bei etwa 18%.^3^ Aufgrund des Risikos, in ein invasives Karzinom überzugehen, sind eine frühzeitige Diagnose und Behandlung der AK besonders wichtig.

AKs werden klinisch üblicherweise nach Olsen (Grad I–III) und histologisch (keratinozytäre intraepidermale Neoplasie [KIN] I–III) nach einem dreistufigen System klassifiziert.^4^, ^5^ Die Einteilung nach KIN I–III basiert auf der Ausbreitung der atypischen Keratinozyten in der Epidermis. Bei KIN I sind diese Zellen auf das untere Drittel der Epidermis beschränkt. KIN II dagegen zeigt atypische Keratinozyten in den unteren zwei Epidermisdritteln und KIN III in allen Epidermisschichten.^5^, ^6^, ^7^ Ursprünglich wurde angenommen, dass sich AK in einem festen Ablauf über die drei KIN‐Stadien zu cSCC entwickeln. Entsprechend wurde KIN I lange Zeit ein geringes Progressionsrisiko zugeschrieben. Neue Studien zeigen jedoch, dass ein cSCC auch aus niedriggradigen AK hervorgehen kann. KIN I‐Läsionen wurden dabei am häufigsten als direkte Vorstufe eines kutanen Plattenepithelkarzinoms identifiziert.^6^, ^8^ Zwar verbleibt ein Großteil der AK im bestehenden Stadium oder bildet sich sogar zurück, das individuelle Progressionsrisiko ist jedoch nicht vorhersagbar. Aus diesem Grund empfehlen nationale und internationale Leitlinien die konsequente Überwachung und Behandlung aller AK unabhängig vom Schweregrad.^9^, ^10^

Für die Behandlung der AK stehen zahlreiche Behandlungsansätze zur Verfügung. Sie reichen von physikalischen Maßnahmen wie Kryotherapie und photodynamischer Therapie über ablative Verfahren bis hin zu topischen Wirkstoffen.^1^, ^11^ Tirbanibulin‐1%‐Salbe zur topischen Behandlung der AK wurde im Juli 2021 von der *Europäischen Arzneimittelagentur* und im Dezember 2020 von der *US‐amerikanischen Food and Drug Administration* zugelassen.^12^, ^13^ Tirbanibulin ist indiziert zur Feldbehandlung von nicht‐hyperkeratotischer, nicht‐hypertropher AK (Olsen Grad I) im Gesicht oder auf der Kopfhaut bei Erwachsenen. Im Unterschied zu anderen topischen Therapien ist das Behandlungsregime mit Tirbanibulin deutlich kürzer. Es wird lediglich einmal täglich über einen Zeitraum von 5 aufeinanderfolgenden Tagen angewendet. Die Verträglichkeit und Wirksamkeit von Tirbanibulin zur Reduktion von AK wurde in einer Phase‐I‐Studie (NCT02337205), einer Phase‐IIa‐Studie (NCT02838628) und zwei doppelblinden, placebokontrollierten, randomisierten Phase‐III‐Studien (NCT03285477 und NCT03285490) nachgewiesen. Die häufigsten behandlungsbedingten Nebenwirkungen waren leicht ausgeprägt und vorübergehend.^14^ Die Behandlung mit Tirbanibulin führte bei 44% der Patienten in der ersten Phase‐III‐Studie und bei 54% der Patienten in der zweiten Phase‐III‐Studie zu vollständiger Abheilung der Läsionen nach zwei Monaten.^3^ Unter der Behandlung mit Tirbanibulin wurde bis zum Tag 57 die Zahl der AK um 76% (Studie 1) beziehungsweise 82% (Studie 2) reduziert.^3^, ^14^ In diesen Studien wurde Tirbanibulin einmal täglich an fünf aufeinanderfolgenden Tagen auf eine Hautfläche von bis zu 25 cm^2^ im Gesicht oder auf der Kopfhaut aufgetragen. Zwei neuere US‐amerikanische Studien untersuchten die Sicherheit und Verträglichkeit von Tirbanibulin auf einer deutlich größeren Fläche von etwa 100 cm^2^.^15^, ^16^ Auf Grundlage dieser Daten erteilte die FDA die Zulassungserweiterung für die Anwendung von Tirbanibulin auf Flächen bis zu 100 cm^2^.

Die oben genannten klinischen Studien zur Wirksamkeit und Sicherheit von Tirbanibulin basieren auf einer ausgewählten Patientengruppe mit klar definierten Einschlusskriterien. Daher ist ihre Aussagekraft für die Beurteilung der Behandlung unter Alltagsbedingungen begrenzt. Um ein genaueres Bild von der Wirksamkeit und dem Nutzen von Therapien im Praxisalltag besser einschätzen zu können, bieten nichtinterventionelle Studien (NIS) eine wertvolle Ergänzung. Für Tirbanibulin liegen bereits Wirksamkeits‐ und Sicherheitsdaten aus zwei kleineren Beobachtungsstudien (33 und 38 Patienten) und einer retrospektiven Analyse mit 250 Patienten vor.^17^, ^18^, ^19^ Eine Tirbanibulin‐Studie mit 290 Patienten zur medizinischen Versorgung im Praxisalltag in den USA zeigte gute Behandlungsergebnisse, eine verbesserte Lebensqualität und eine hohe Zufriedenheit mit der Behandlung.^20^

Aufgrund der begrenzten Patientenzahl und fehlenden *Real‐World*‐Daten zu Patienten mit AK in Deutschland war das Ziel dieser Studie, Daten zur Wirksamkeit und Sicherheit von Tirbanibulin sowie zu patientenberichteten Ergebnissen (*Patient Reported Outcomes*, PROs) in einer großen, überwiegend ambulant behandelten Patientenkohorte in Deutschland zu erheben und auszuwerten.

## PATIENTEN UND METHODIK

### Studiendesign und ‐setting

Bei der KLIR‐Studie (Klisyri^®^ in *Real World Treatment*) handelt es sich um eine prospektive, offene, multizentrische NIS, die zwischen Februar 2022 und September 2023 an 58 dermatologischen Kliniken und Praxen in Deutschland durchgeführt wurde. Vor Studienbeginn wurden alle relevanten Unterlagen von der Ethikkommission der Friedrich‐Alexander‐Universität Erlangen‐Nürnberg geprüft (Nr.: 21‐444‐NIS; positives Votum vom 15. Dezember 2021). Die Datenerhebung erfolgte in Übereinstimmung mit der aktuellen Fassung der Deklaration von Helsinki sowie den geltenden Richtlinien und Empfehlungen zur Guten Epidemiologischen Praxis. Die Verordnung der Tirbanibulin‐1%‐Salbe, der Besuchsplan und die Datenerfassung während dieser NIS entsprachen der klinischen Routineversorgung in Deutschland.

Der Zeitraum für die Beurteilung des Ansprechens umfasste drei Visiten: Visite 1 als Ausgangswert, Visite 2 zwischen Tag 8–29 (optional) und Visite 3 an Tag 57. Die Gesamtdauer der Studie betrug daher etwa 8 Wochen (abhängig von den tatsächlichen Patiententerminen). Dies entspricht den Behandlungsempfehlungen und der vorgesehenen Behandlungsdauer, wie sie in der Fachinformation angegeben sind. Es folgte ein optionale langfristige Nachbeobachtung von circa 6 Monaten (Visite 4, geplant an Tag 240), so dass sich eine Gesamtdokumentationszeit von circa 8 Monaten pro Patient ergab.

### Teilnehmer

In die Studie eingeschlossen wurden erwachsene Patienten (≥ 18 Jahre) mit AK, die gemäß der Fachinformation für eine Behandlung mit Tirbanibulin‐1%‐Salbe geeignet waren und ihre Einverständniserklärung abgegeben haben.

### Durchführung und Auswertung

**ABBILDUNG 1:**
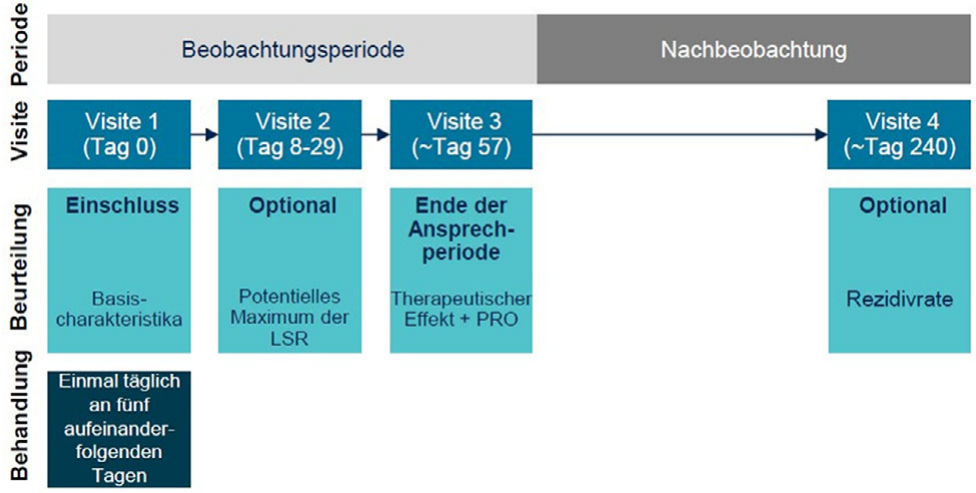
Behandlungs‐ und Visitenplan von KLIR.

Abbildung 1 zeigt den Behandlungsplan und die geplanten Patientenvisiten der KLIR‐Studie. Die Aufnahme in die Studie erfolgte bei der ersten Visite. Zu den Basisdaten zählten der Fitzpatrick‐Hauttyp, die Anzahl und Lokalisation der Läsionen im Behandlungsareal (5 x 5 cm) sowie frühere AK‐Behandlungen innerhalb der letzten 12 Monate (gemäß *Case Report Form*). Tirbanibulin 10 mg/g (1%ige Salbe) sollte gemäß der Fachinformation angewendet werden. Eines der primären Ziele war die Evaluation der Sicherheit und Verträglichkeit von Tirbanibulin, dokumentiert anhand von unerwünschten Ereignissen. Dazu zählten unerwünschte Arzneimittelwirkungen und unerwünschte Ereignisse von besonderem Interesse (*adverse event of special interest*; AESI) im Behandlungsbereich bei allen Patienten, die mindestens eine Dosis Tirbanibulin erhalten hatten. Unerwünschte Ereignisse von besonderem Interesse wurden definiert als Hauttumore, die im Behandlungsbereich auftraten. Lokale Hautreaktionen (*local skin reactions*; LSR) wurden getrennt von den oben genannten sicherheitsrelevanten Ereignissen dokumentiert. Unerwünschte Ereignisse und LSR wurden während der gesamten Studie kontinuierlich erfasst. Darüber hinaus wurde ein mögliches Maximum der LSR während der optionalen Visite 2 ermittelt. Die therapeutische Wirksamkeit wurde anhand der Reduktion der AK‐Läsionen und der Abheilungsraten (vollständige (100%ige Reduktion der Läsionen) und partielle Abheilung (Reduktion der Läsionen um ≥ 75%)) bei Visite 3 im Vergleich zum Ausgangswert beurteilt. Zusätzlich wurden bei Visite 3 die teilnehmenden Ärzte, Patienten und deren Partner über ihre Zufriedenheit mit der Behandlung befragt (zusätzliche Endpunkte). Die Zufriedenheit der Ärzte wurde anhand einer 4‐Punkte‐Skala ermittelt (sehr zufrieden, zufrieden, unzufrieden, sehr unzufrieden). Zur Erfassung der PROs wurden standardisierte und routinemäßig eingesetzte Fragebögen verwendet, darunter der *Patient Global Improvement Index* (PGII),^21^ eine Skala zur Bewertung des kosmetischen Ergebnisses und ein Fragebogen zur zukünftigen Behandlungspräferenz. Mit Hilfe des PGIII bewerteten die Patienten ihren Krankheitszustand im Vergleich zum Ausgangswert auf einer 7‐Punkte‐Skala (vollständig gebessert [geheilt], deutlich gebessert, moderat gebessert, leicht gebessert, keine Veränderung, leicht verschlechtert, deutlich verschlechtert). Das kosmetische Ergebnis bewerteten die Patienten und deren Partner auf einer 4‐Punkte‐Skala (stark verbessert, etwas verbessert, keine Veränderung, verschlechtert). Die zukünftige Behandlungspräferenz (*Patient's Future Treatment Preference*) der Patienten, das heißt die Bereitschaft zur Wiederholung der Tirbanibulintherapie, wurde mit Hilfe einer 5‐Punkte‐Skala erfasst (auf jeden Fall, sicherlich, wahrscheinlich, vielleicht, auf keinen Fall). Die Therapieadhärenz wurde anhand der Anwendungstage von Tirbanibulin bewertet. Bei der langfristigen Nachbeobachtung (Visite 4) wurden Sicherheit und Rezidivrate (zusätzlicher Endpunkt) ermittelt. Dazu wurde das Wiederauftreten oder die Entwicklung neuer AK‐Läsionen im Behandlungsgebiet bei Patienten, die bei Visite 3 eine vollständige Abheilung erreicht hatten, bestimmt. Mit Hilfe von papierbasierten Fallberichtsformularen und PRO‐Formularen wurden die Daten für jeden Patienten erhoben.

### Statistische Auswertung

Die statistischen Analysen wurden mit deskriptiven Methoden durchgeführt. Ein Beobachtungsfallansatz wurde für die tabellarische Darstellung der Daten nach Visiten verwendet. Kontinuierliche Variablen wurden durch die Anzahl der nicht fehlenden Werte, die Anzahl der fehlenden Werte, den arithmetischen Mittelwert, die Standardabweichung, den Median, das Minimum und das Maximum beschrieben. Für jede beobachtete Modalität wurden die kategorialen Variablen als absolute Häufigkeit und als relativer Prozentsatz dargestellt. Die Abheilungsraten wurden als Prozentsatz pro Visite mit exakten 95%‐Clopper‐Pearson‐Konfidenzintervallen (KI) berechnet. Sofern nicht anders angegeben, wurden zweiseitige 95%‐Konfidenzintervalle abgeleitet. Die p‐Werte waren ebenfalls zweiseitig und wurden mit einem Signifikanzniveau von 5% interpretiert. Eine Subgruppenanalyse der Abheilungsraten wurde für Patienten mit Visite 3 zwischen Tag 47 und 67 durchgeführt.

Da es sich um eine NIS handelte, entfiel eine formale Stichprobenberechnung. Im Allgemeinen wurden fehlende Werte nicht imputiert. Die Daten wurden so analysiert, wie sie in der klinischen Datenbank vorlagen, mit Ausnahme unvollständiger Abbruchdaten für die AK‐Diagnose, frühere AK‐Behandlungen und Begleitmedikation. Bei fehlenden Tagen wurde der letzte Tag des Monats zugrunde gelegt. Andernfalls wurde nicht imputiert und das Datum wurde als fehlend behandelt.

Die statistische Analyse dieser Studie wurde mit der SAS^®^‐Software Version 9.4 (SAS Institute Inc., Cary, NC, USA) durchgeführt.

## ERGEBNISSE

### Patientendisposition, Demografie und Basisdaten

Insgesamt wurden 545 Patienten in die Studie eingeschlossen. Davon wurden zwei Patienten nicht behandelt und ein Patient erfüllte die Einschlusskriterien nicht. Die Sicherheits‐ und Wirksamkeitspopulation setzte sich somit aus 543 beziehungsweise 542 Patienten zusammen. An der optionalen zweiten Visite zur Beurteilung eines möglichen Maximums der LSR nahmen 516 Patienten nach durchschnittlich 21,7 Tagen (± 20,7 Tagen) teil. Nach durchschnittlich 72,4 Tagen (± 25,3 Tage) wurde für 507 Patienten das Ansprechen (Visite 3) dokumentiert. Bei der optionalen Visite 4 zur Beurteilung des AK‐Rezidivs wurden insgesamt 437 Patienten untersucht.

**ABBILDUNG 2:**
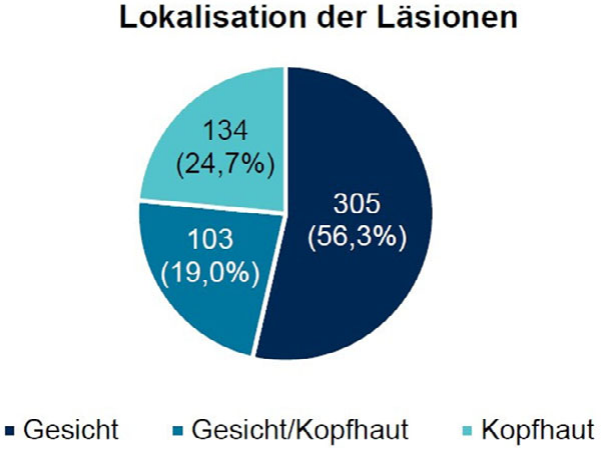
Lokalisation der AK‐Läsionen bei Studienbeginn (n = 542).

Das mediane Alter der Patienten bei Studienstart betrug 74 Jahre (40–99 Jahre), 67,8% waren männlich (Tabelle 1), und die Mehrheit wiesen die Fitzpatrick‐Hauttypen II (52,1%) und III (22,7%) auf. Im Behandlungsbereich wurden durchschnittlich 5,9 (± 4,6) AK‐Läsionen beobachtet, überwiegend im Gesicht (n = 305; 56,3%), seltener auf der Kopfhaut (n = 103; 24,7%) und in beiden Bereichen (n = 130; 19,0%) (Abbildung 2). Mehr als die Hälfte der Patienten hatte in den 12 Monaten vor der Studie mindestens eine vorangegangene Therapie gegen AK erhalten (n = 298; 54,9%). Am häufigsten wurden topische medikamentöse Therapien eingesetzt (n = 158; 29,1%), gefolgt von Kryotherapie (n = 129; 23,8%) (Tabelle 1). Patienten mit Immunsuppression wurden nicht von der Studie ausgeschlossen. Unter den Studienteilnehmenden waren insgesamt zwölf Patienten mit Immunsuppressiva und zwei Patienten mit hämatologischen Malignomen (akute myeloische Leukämie [AML] und chronische lymphatische Leukämie [CLL]). Die mediane Behandlungsdauer mit Tirbanibulin betrug 5 Tage (1–8 Tage) und 90,3% der Patienten (437 von 484 Patienten [beobachtete Fälle]) erhielten Tirbanibulin fünfmal.

**TABELLE 1:** Demografie und Basisdaten.

Parameter	n = 543
***Demographische Daten***	
Alter (Jahre), Median (Spanne)	74,0 (40‐99)
Männlich, n (%)	368 (67,8)
Anzahl der klinisch beurteilten AK‐Läsionen, Mittelwert (SD)	5,9 (±4,6)
***Vorherige AK‐Therapie, n (%)***	
Ja	298 (54,9)
Nein	239 (44,0)
Vorangegangene AK‐Therapie^*^	
Chemisches Peeling	32 (5,9)
Kryotherapie	129 (23,8)
Laserablation	6 (1,1)
Photodynamische Therapie	45 (8,3)
Chirurgischer Eingriff	7 (1,3)
Systemische medikamentöse Therapie	2 (0,4)
Topische medikamentöse Therapie	158 (29,1)
Andere Therapie	3 (0,6)

*Abk*.: n, Anzahl der Patienten in der Analysepopulation; n, Anzahl der Patienten unter n; (%), prozentualer Anteil unter n; SD, Standardabweichung

* Mehrfachnennungen waren möglich.

### Wirksamkeit

**ABBILDUNG 3:**
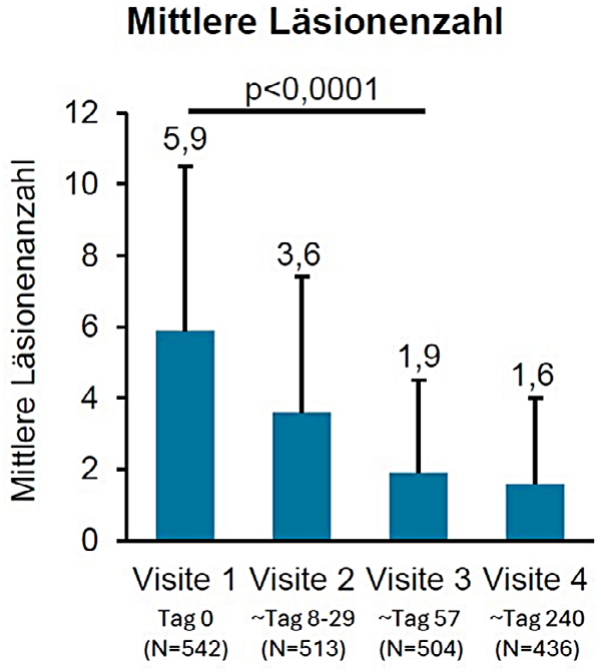
Mittlere Anzahl an AK‐Läsionen und SD bei Studienbeginn (Visite 1 [n = 542]), bei Visite 2 (n = 513), bei Visite 3 (n = 504) und bei Visite 4 (n = 436).

Die durchschnittliche Anzahl der AK‐Läsionen sank von 5,9 (±4,6) bei Studienbeginn auf 1,9 (± 2,6) am Ende des Bewertungszeitraums (Visite 3), was einer mittleren Reduktion von 4,1 Läsionen entspricht (95%‐KI: –4.5 bis –3.8). Bei Visite 4 betrug die durchschnittliche Anzahl der Läsionen 1,6 (± 2,4) und kommt damit einer Reduktion von 4,5 Läsionen im Vergleich zum Ausgangswert gleich (Abbildung 3). Insgesamt wurde eine signifikante Reduktion der AK‐Läsionen festgestellt: im Durchschnitt um 70% (*p* < 0,0001) bis zur Visite 3 nach etwa 72,4 Tagen und um 73% bis zur Visite 4 nach rund 240 Tagen, jeweils im Vergleich zum Ausgangswert.

Die Abheilungsraten wurden am Ende der Studie (laut Plan: Tag 57) bestimmt. Da es sich bei der Studie um ein nichtinterventionelles Design handelte, gab es keinen festgelegten Besuchsplan. Die dritte Visite fand bei den Teilnehmenden zwischen Tag 26 und 248 statt. Aus diesem Grund wurden die Abheilungsraten getrennt berechnet: einmal für die gesamte Patientengruppe (n = 505) und für eine Subgruppe (n = 277). Diese Subgruppe entsprach den Vorgaben der Fachinformation und umfasste nur Patienten, bei denen Visite 3 zwischen Tag 47 und Tag 67 (Tag 57 ± 10 Tage) stattfand. In der gesamten Patientengruppe erreichten 189 von 505 Patienten (37,4%; 95%‐KI: 33,2–41,8) eine vollständige Abheilung der Läsionen im Vergleich zum Ausgangswert. Bei insgesamt 278 Patienten (55,0%; 95%‐KI: 51,6–60,5) wurde eine partielle Abheilung beobachtet, definiert als Rückgang der Läsionsanzahl um ≥ 75 % gegenüber dem Ausgangswert. In der Subgruppe der Patienten, bei denen Visite 3 zwischen Tag 47 und Tag 67 stattfand (n = 277), zeigten 127 (45,8%; 95%‐KI: 39,9–51,9) eine vollständige und 178 (64,3%; 95%‐KI 58,3–69,9) eine partielle Abheilung.

Die Rezidivrate der AK‐Läsionen im Behandlungsbereich betrug nach der langfristigen Nachbeobachtung (Visite 4) 21,9% (34 von 155 der Patienten, die bei Visite 3 eine vollständige Abheilung aufwiesen).

### Sicherheit und Verträglichkeit

Insgesamt wurden 23 unerwünschte Ereignisse bei 13 Patienten (2,4%) berichtet (Tabelle 2). Davon waren drei schwerwiegende unerwünschte Ereignisse (n = 2 [0,4%]), von denen keines als behandlungsbedingt eingestuft wurde. Ein Patient wurde wegen Fieber stationär aufgenommen, ein weiterer Patient musste einmal wegen einer Herzkatheteruntersuchung und einmal wegen eines Herzschrittmacherwechsels stationär behandelt werden. Keine der unerwünschten Ereignisse führte zu Dosisänderungen, zum Studienabbruch oder zum Behandlungsabbruch mit Tirbanibulin. Zudem wurden keine AESI beobachtet (Tabelle 2). Etwa die Hälfte der unerwünschten Ereignisse wurden als unerwünschte Arzneimittelwirkungen eingestuft (12 Ereignisse bei 4 Patienten). Die häufigsten dokumentierten unerwünschte Arzneimittelwirkungen standen im Zusammenhang mit Haut‐ und Weichteilgewebserkrankungen und betrafen drei Patienten (0,6%) (Tabelle 2). Zu den Symptomen zählten unter anderem Erytheme, atopische Dermatitis und Schuppung der Haut. Alle Ereignisse dieser Kategorie waren von leichtem Schweregrad, mit Ausnahme eines Falles mit mittelschwerem Erythem.

**TABELLE 2:** Unerwünschte Ereignisse (Mehrfachnennungen möglich).

n (%)	n = 543	
	n (%)	Ereignisse
Unerwünschte Ereignisse (UE) insgesamt	13 (2,4)	23
Schwerwiegendes unerwünschtes Ereignis	2 (0,4)	3
Vorzeitiger Studienabbruch aufgrund von UE	0 (0,0)	0
Vorzeitiger Behandlungsabbruch aufgrund von UE	0 (0,0)	0
Unerwünschte Arzneimittelwirkung	4 (0,7)	12
Unerwünschtes Ereignis von besonderem Interesse (AESI)	0 (0,0)	0
***Unerwünschte Arzneimittelwirkungen*** ^*^		
Augenerkrankungen	2 (0,4)	2
Allergische Konjunktivitis	1 (0,2)	1
Augenirritation	1 (0,2)	1
Gastrointestinale Erkrankungen	1 (0,2)	1
Stomatitis	1 (0,2)	1
Allgemeine Erkrankungen und Reaktionen an der Applikationsstelle	2 (0,4)	2
Grippeähnliche Erkrankung	1 (0,2)	1
Schwellung	1 (0,2)	1
Atemweg‐, Brustkorb‐ und Mediastinumerkrankungen	1 (0,2)	1
Nasentrockenheit	1 (0,2)	1
Haut‐ und Weichteilgewebserkrankungen	3 (0,6)	6
Atopische Dermatitis	1 (0,2)	1
Erythem	2 (0,4)	3
Schuppung der Haut	1 (0,2)	2

*Abk*.: n, Anzahl der Patienten in der Analysepopulation; n, Anzahl der Patienten unter n; [Ereignisse], Anzahl der einzelnen Ereignisse, die unter den n Patienten auftraten.

* Mehrfachnennungen waren möglich

Die LSR wurden getrennt von den oben genannten sicherheitsrelevanten Ereignissen dokumentiert. Bei 538 von 543 Patienten (99,1%) wurde mindestens ein LSR dokumentiert (insgesamt bei allen Visiten), von denen jedoch keines als schwerwiegend eingestuft wurde. Die häufigsten LSR waren „Erythem“ (97,6%) und „Schuppung“ (90,0%), gefolgt von „Verkrustung“ (63,6%), „Schwellung“ (20,8%), „Erosion/Ulkus“ (17,1%) und „Bläschen/Pusteln“ (8,9%). Die meisten LSR waren von leichtem (65,4%) und mittlerem (29,5%) Schweregrad.

### Zufriedenheit der Ärzte sowie patientenberichtete Ergebnisse (PROs)

**ABBILDUNG 4:**
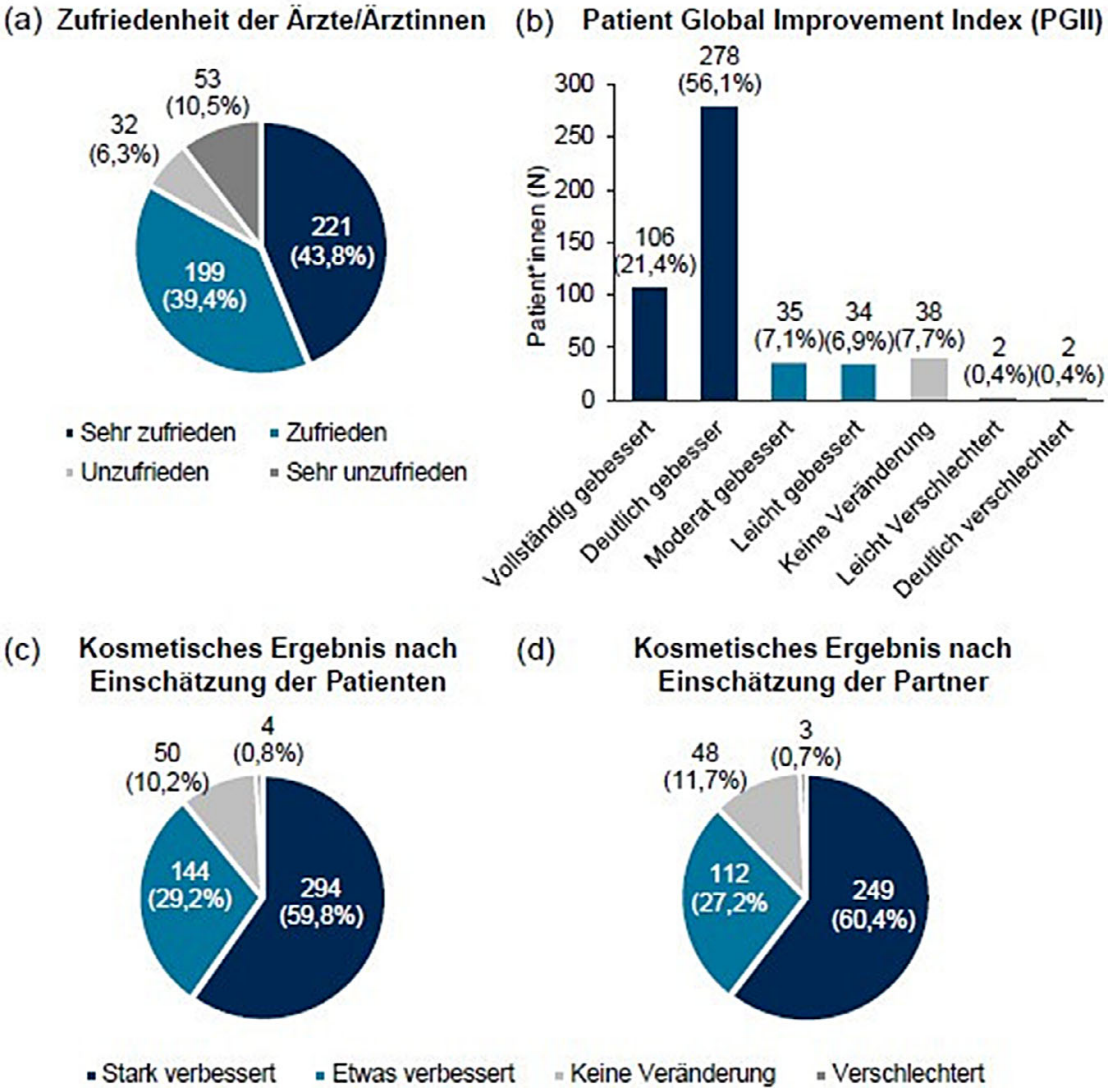
(a) Zufriedenheit der teilnehmenden Ärzte mit dem Behandlungsergebnis (n = 505), (b) Patientenzufriedenheit in Bezug auf die Verbesserung des Krankheitsbildes und der Symptome (Patient Global Improvement Index) (n = 495), (c) Zufriedenheit mit dem kosmetischen Ergebnis nach Einschätzung der Patienten (n = 492) und (d) Zufriedenheit mit dem kosmetischen Ergebnis nach Einschätzung der Partner (n = 412) bei Visite 3 (circa Tag 57).

Die teilnehmenden Ärzte waren am Ende der Beobachtungsperiode mit dem Behandlungsergebnis von 83,2% (420 von 505) der Patienten zufrieden („sehr zufrieden“ (43,8%) und „zufrieden“ (39,4%)) (Abbildung 4a).

Insgesamt bewerteten 91,5% der Patienten (453 von 495 Patienten) ihre AK‐Läsionen und die damit verbundenen Symptome bei Visite 3 als verbessert. Verglichen mit der Ausgangslage bewerteten 77,5% der Patienten ihre Symptome als „vollständig gebessert“ und „deutlich gebessert“, 14,0% als „moderat gebessert“ und „leicht gebessert“. „Keine Veränderung“ beziehungsweise eine Verschlechterung („leichte“ und „deutliche“ Verschlechterung) der Symptome verglichen mit dem Zustand vor der Behandlung wurde von 7,7% beziehungsweise 0,8% der Patienten angegeben (Abbildung 4b). Dementsprechend wurde das kosmetische Ergebnis von 89,0% der Patienten und 87,6% ihrer Partner als verbessert beurteilt (Abbildung 4c,d).

**ABBILDUNG 5:**
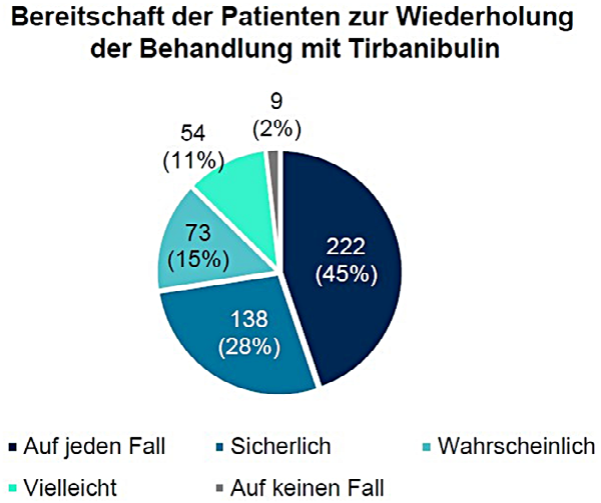
Bereitschaft der Patienten zur Wiederholung der Behandlung mit Tirbanibulin (n = 496) bei Visite 3 (circa Tag 57). Aufgrund von Rundungen kann die Summe der Prozentsätze mehr als 100 % betragen.

Bezüglich der zukünftigen Behandlungspräferenzen gaben fast alle Patienten (99%) an, Tirbanibulin zur Behandlung von AK wieder in Betracht zu ziehen (45% auf jeden Fall, 28% sicherlich, 15% wahrscheinlich, 11% vielleicht) (Abbildung 5).

## DISKUSSION

Diese NIS mit einer großen, multizentrischen Kohorte von fast 550 Patienten lieferte wesentliche Erkenntnisse zur Wirksamkeit, Sicherheit und zu den PROs der topischen Anwendung von Tirbanibulin 1% bei AK in Deutschland. Die Ergebnisse zeigen, dass Tirbanibulin die Läsionszahl deutlich reduzieren konnte. Dies spiegelte sich auch in der hohen Zufriedenheit sowohl der Patienten als auch der behandelnden Ärzte wider, sowie in der großen Anzahl von Teilnehmenden, die angaben, Tirbanibulin auch künftig zur Behandlung von AK in Betracht zu ziehen.

Zur Behandlung der AK gibt es verschiedene Therapiemöglichkeiten, darunter auch topische Medikamente, die einfach und komfortabel in der Anwendung sind.^13^ Viele dieser topischen Therapien erfordern jedoch eine längere Behandlungsdauer (Diclofenac 60–90 Tage, 5‐Fluorouracil 4–12 Wochen, Imiquimod zwei 4‐wöchige Zyklen).^7^ Im Gegensatz dazu muss die 1%ige Tirbanibulinsalbe nur einmal täglich über einen Zeitraum von 5 aufeinanderfolgenden Tagen angewendet werden.^3^, ^18^ Die kurze Behandlungsdauer könnte ein wichtiger Faktor für die hohe Adhärenzrate von über 90% gewesen sein. Auch eine frühere Praxisstudie mit Tirbanibulin zeigte, dass fast 90% der Patienten ihre Therapietreue mit Tirbanibulin als „gut“ oder „sehr gut“ einschätzten. Diese hohe Adhärenz könnte zur in dieser Studie beobachteten wirksamen Läsionsreduktion beigetragen haben (Reduktion der Gesamtzahl der Läsionen um 70% nach durchschnittlich 72,4 Tagen und um 73% bei Besuch 4 im Vergleich zum Ausgangswert). Insgesamt lagen die Abheilungsraten dieser Studie in einem ähnlichen Bereich wie die Ergebnisse aus Phase‐II‐ und Phase‐III‐Studien sowie einer weiteren praxisnahen Untersuchung.^3^, ^14^, ^17^

Besonders hervorzuheben ist, dass im behandelten Hautareal keine neuen Hauttumoren beobachtet wurden. Die gemeldeten unerwünschten Ereignisse und LSR entsprechen dem bereits bekannten Sicherheitsprofil und bestätigen erneut die gute Verträglichkeit und Sicherheit von Tirbanibulin.^3^, ^14^, ^19^ Der Wirkmechanismus des Proliferationsinhibitors Tirbanibulin beruht auf der Hemmung der Tubulinpolymerisation, was zur Apoptose krankhafter Zellen führt.^22^, ^23^ Die reversible Tirbanibulinbindung könnte die in klinischen Studien überwiegend beobachteten leichten bis mittelschweren LSR erklären. Dies wiederum kann die Therapietreue und die Bereitschaft der Patienten erhöhen, die Behandlung fortzusetzen oder zu wiederholen.

Neben den klinischen Daten liefern PROs wichtige Einblicke in die Behandlung aus Sicht der Patienten. Die Ergebnisse zeigten, dass die Mehrheit mit der Behandlung zufrieden war und insbesondere das kosmetische Ergebnis von vielen Patienten und ihren Partnern als verbessert wahrgenommen wurde. Nahezu alle (99%) gaben an, sich eine erneute Behandlung mit Tirbanibulin vorstellen zu können, was auf eine hohe Akzeptanz dieser Therapie hinweist. Diese Beobachtungen stimmen mit den Ergebnissen der nichtinterventionellen PROAK‐Studie aus den USA überein, in der ebenfalls PROs erhoben wurden. Auch dort zeigte sich eine hohe Zufriedenheit mit der Behandlung und eine große Bereitschaft von Patienten sowie Ärzte, Tirbanibulin künftig erneut zur Behandlung von AK einzusetzen.^20^

Da diese Studie ausschließlich in Deutschland durchgeführt wurde, ist die Übertragbarkeit der Ergebnisse auf andere Länder möglicherweise eingeschränkt. Da es sich um eine nichtinterventionelle Studie ohne Vergleichsgruppe handelt, sind die Möglichkeiten, klare Ursache‐Wirkungs‐Zusammenhänge herzustellen, eingeschränkt. Es kann nicht ausgeschlossen werden, dass es bei der Patientenauswahl zu Unausgewogenheiten kam, etwa weil eher Personen teilnahmen, die generell offen für Forschungsstudien sind. Gleichzeitig ist hervorzuheben, dass alle erfassten Parameter (zum Beispiel Läsionszahlen, LSR und PROs) Teil der klinischen Routineversorgung bei AK in Deutschland sind. Das nichtinterventionelle Studiendesign ermöglichte die Erhebung von Daten über ein breites Besuchsfenster hinweg. Dadurch wurden die Visiten 2, 3 und 4 jedoch zu unterschiedlichen Zeitpunkten dokumentiert, was die Vergleichbarkeit der Ergebnisse einschränkt. Zudem nahm die Zahl der Patienten, die im Verlauf zur Nachsorge erschienen, ab, was auf eine mögliche Verzerrung, zum Beispiel im Zusammenhang mit Therapieerfolg oder Therapiemisserfolg, hindeutet. Nichtsdestotrotz war die Gesamtteilnahmequote über die Studiendauer hinweg hoch.

Zusammenfassend bestätigen die Studienergebnisse, dass Tirbanibulin unter Alltagsbedingungen sowohl wirksam als auch gut verträglich ist. Darüber hinaus verdeutlichen die Daten, dass Patienten die Behandlung mit Tirbanibulin positiv bewerten und die topische Behandlung sehr schätzen. Mit Blick auf die Zukunft zielen laufende und geplante groß angelegte Studien darauf ab, die wissenschaftliche Grundlage für den Einsatz von Tirbanibulin weiter zu stärken. Dazu gehören eine dreijährige Sicherheitsstudie im direkten Vergleich mit Diclofenac,^24^ Untersuchungen zur Anwendung von Tirbanibulin bei immunsupprimierten Patienten, sowie eine umfassende europäische Studie zur Bewertung der Sicherheit und Wirksamkeit von Tirbanibulin bei der Behandlung größerer Hautareale.^25^

## DANKSAGUNG UND FINANZIERUNG

Die Autoren danken den beteiligten Studienzentren und ‐teams herzlich für ihre wertvollen Beiträge zu dieser Arbeit. Die Zentren erhielten eine Aufwandsentschädigung, abhängig von der Anzahl der dokumentierten Patienten. Die Erstellung des Manuskripts wurde durch *Medical Writing* von Dr. Alexandra Ehrens, med:unit GmbH, Deutschland, unterstützt und durch Almirall Hermal GmbH, Reinbek, Deutschland, finanziert. Die Autoren behielten dabei jederzeit die volle inhaltliche Kontrolle und haben der finalen Version des Manuskripts vollständig zugestimmt.

Open access Veröffentlichung ermöglicht und organisiert durch Projekt DEAL.

## INTERESSENKONFLIKT

M.V.H. war Mitglied in Fachbeiräten von Almirall Hermal, Sanofi‐Aventis, MSD, Bristol‐Myers Squibb und Novartis und erhielt Referentenhonorare von Galderma und Biofrontera. I.H. war als Beraterin und/oder Referentin für AbbVie, Almirall, Galderma, Janssen, Leo Pharma, Novartis, Pfizer, Regeneron, Sanofi und UCB Pharma tätig. A.K. und A.M. sind Mitarbeiterinnen der Almirall Hermal GmbH. C.B. erhielt Referentenhonorare von Almirall Hermal, Bristol Myers Squibb, Novartis, Merck Sharp & Dohme, Regeneron und Leo Pharma sowie Beraterhonorare von Delcath, Immunocore, Bristol Myers Squibb, Novartis, Merck Sharp & Dohme, Almirall Hermal, Pierre Fabre, Sanofi, Regeneron und SkylineDx.
